# Migration outside large cities: a comparison of the hiring of migrants for the food processing industry in the United States and Japan

**DOI:** 10.1186/s40878-021-00258-w

**Published:** 2021-10-21

**Authors:** Yusuke Mazumi

**Affiliations:** Institute of Liberal Arts and Science, Kanazawa University, Kakuma-machi, Kanazawa, Ishikawa 920-1192 Japan

**Keywords:** Labor migration, New destinations, Industrial restructuring, Food processing industry, Japan, The United States

## Abstract

Recent studies suggest that the hiring of migrants in the food processing industry has increased the migrant population outside large cities among affluent migrant-receiving countries. This study examines how the U.S. meatpacking industry and the Japanese seafood processing industry, in particular, have developed a dependence on migrants; it does so to identify whether and how a common—thus cross-nationally generalizable—process may account for migration outside large cities. A comparative historical analysis revealed that, with significant national differences between the United States and Japan, including in the legal and institutional contexts of migration, there is little commonality in the processes through which the industries have come to depend on migrants. Yet, there is a similarity in the development of mass production. Such production necessitates an undisrupted availability of full-time as well as low-wage workforce, and migrants on both sides of the Pacific are employed to ensure this availability. Thus, while urban-centered migration studies often emphasize the growth of low-wage services or small-batch manufacturing as an economic driver of migration, this study argues that, outside large cities, a different pattern of industrial transformation is associated with labor migration.

## Introduction

Labor migration has long been an urban phenomenon in many affluent migrant-receiving countries. Yet, recent studies have documented the growth of the migrant population outside large cities as well (Hugo & Morén-Alegret, 2008; Jentsch & Simard, 2009; McAreavey & Argent, 2018; Rye & Scott, 2018). One important reason is the hiring of migrants in the food processing industry, and the United States is a notable example. Being the largest manufacturing employer in rural America (Rural Migration News, 2011), since the 1990s, the meatpacking industry has actively employed Mexican and other Latinx migrants in rural Southern and Midwestern regions, where their population had traditionally been scarce (Kandel & Parrado, 2005; Marrow, 2011; Stuesse, 2016; Stull & Broadway, 2004). Migrants have recently been shown to comprise 37% of the total meat processing industrial workforce (Migration Policy Institute, 2020), a number which is more than double the number of foreign-born individuals in the country. This migration partly contributes to the emergence of what the U.S. immigration literature now calls Latinx migrants’ “new destinations” (Flippen & Farrell-Bryan, 2021; Zúñiga & Hernández-León, 2005).

Though less often noted, studies also find a new link between the hiring of migrants in the food processing industry and populational increase outside large cities in other countries, including Canada (Broadway, 2007), Norway (Rye, 2018), and the United Kingdom (Lever & Milbourne, 2017; Tannock, 2015). Japan offers still another case, where this industry alone employs almost 8% of all migrant workers in the country.^1^ Particularly relevant is the seafood processing sector, one of the largest food processing sectors in this fish-eating nation, which has increasingly employed migrants in the last 20 years. Of the total of 171,354 persons working in this sector in 2018, 10% were migrants,^2^ which is a significant figure given the average share of Japan’s foreign population (a little over 2%). This hiring has contributed to the increasing number of migrants in coastal seafood-processing cities and towns (Mazumi, 2019; Miki, 2005; Sasaki, 2014).

Focusing on the United States and Japan, this study examines how, during the last few decades, the food processing industry has come to depend on migrants. It does so to explore whether and, if so, how a common—thus cross-nationally generalizable—process exists that accounts for this dependence between the countries. Previous studies tend to investigate a single national case; thus, very little research has delved into this comparative question. Yet addressing this issue is important, given that the hiring of migrants of this industry is a part of common factors that created migration flow into non-large cities among developed countries. To the extent that there is a cross-nationally generalizable process through which the food processing industry has developed its dependence on migrants, identifying such commonality can shed a partial, but important, light on general processes of migration outside large cities among these countries.

Comparing the United States and Japan has a strategic significance for the aim of this study. Although the food processing industry in both countries has actively hired migrants since a more or less similar time period, the conditions under which it has been done are very different. Two differences are noteworthy, including, as elaborated in the following section, legal and institutional contexts of migration, and industrial characteristics of each food processing business (with the U.S. companies being larger and more growing). These differences may lead us to expect very little commonality in the process through which the food processing industry has come to employ migrants between the countries. This study seeks to reveal whether a cross-nationally generalizable process can still be found under these different conditions.

Perhaps not surprisingly, the comparative historical analysis below identifies few similar processes that have led the U.S. and Japanese food processors to hire migrants. There is one exception, however, which is the development of mass production. Though the average production size differs, both the U.S. meatpacking and Japanese seafood processing industries have established mass production in the postwar years, and those food processors that went through this process tend to employ migrants. They are more likely to secure the constant (that is, not seasonal or part-time) availability of low-wage workforce in order for their mass production to work, and migrants are hired to ensure this availability.

The influential global city thesis by Sassen (1988), [1991] 2001) observes the decline of mass manufacturing and the increase of professional service occupations (as in producer services) in world-famous large cities, which, it suggests, in turn led to the growth of low-wage service jobs and “downgraded” manufacturing sectors (notably, small-batch production with labor-intensive work processes, as in sweatshops) that support urban elites; according to the thesis, this restructuring of urban industries relates to labor migration (for statistical assessment of the thesis, see Sanderson et al., 2015). While, currently, migrants also relocate to non-large cities to do low-wage work, the case of the food processing industry indicates that a different pattern of industrial transformation is associated with labor migration there. Specifically, this study finds that the development of mass production produced demand for low-wage jobs in this industry, which led to the hiring of migrants.

## Case selection and logic of comparison

Comparing the United States and Japan is important for this study. First, the United States is significant because its meatpacking industry has created significant migration flow into rural areas. Relocation of Mexican and other Latinx migrants is particularly relevant. While having traditionally concentrated on a handful of gateway states such as California, over the last three decades, these migrants have increased their presence in other parts of the country, especially the South and Midwest (Massey & Capoferro, 2008; Zúñiga & Hernández-León, 2005). They have resided in rural, as well as urban, areas in these regions (Kandel & Cromartie, 2004; Lichter & Johnson, 2020). The growth of job opportunities in the meatpacking industry, which locates its major production sites in these regions, is one factor. Research by Kandel and Parrado (2005), using the Current Population Surveys, finds that, during the 1990s, an increase in the local share of the meatpacking workforce was strongly associated with that in the Latinx share in the local population among non-metropolitan counties.^3^ Case studies also document the growth of the Latinx migrant population in places that host meatpacking facilities, such as Garden City, Kansas (Stull & Broadway, 2004) and Marshalltown, Iowa (Grey & Woodrick, 2002). Furthermore, the hiring of Latinx migrants has continued to date, and this knowledge became indirectly known through the COVID-19 outbreak in several major meatpacking plants (Telford & Kindly, 2020). The report by the Centers for Disease Control and Prevention, released in July, 2020, suggested that, among the 9919 cases reported in the industry with race/ethnicity, 56% were Hispanics (i.e., Latinxs; though this figure include the native-born as well) (Waltenburg et al., 2020). Migrants in the industry include the unauthorized, with estimates varying from 14% to the majority of the total workforce in some plants (Groves & Tareen, 2020).

Second, Japan provides a strategic case to compare with the United States. For one thing, like the U.S. meatpacking counterpart, Japan’s seafood processing industry has depended on migrants outside large cities. Yet, a more important reason is that it has done so despite significant differences in condition. Such differences involve legal and institutional contexts of migration as well as industrial characteristics of the food processing business. These differences make the U.S. and Japanese cases potentially two contrasting ones among those experiencing the similar phenomenon. This contrast is important because, if there is any similarity in the process through which food processors developed their dependence on migrants across countries, such similarity should be identified between contrasting cases as well. Put another way, the Japanese case can serve as a rigorous benchmark to specify the extent to which the processes observed in the United States are generalizable outside its own context.

Specifically speaking, first, legal and institutional contexts of migration, in which businesses hire migrants, are different. Hosting the largest number of migrants in the world, the United States has one of the most expansive contexts. In 2018, there were 44.8 million persons counted as migrants, accounting for 14% of the total population in the country. Of these, 72% were either legal permanent residents or naturalized citizens, and 23% were the unauthorized (Budiman, 2020).

Japan’s context differs significantly. There are existing governmental policies that do not admit the entry of migrants for permanent residency. Additionally, up until 2019, when the new “specified skilled worker” visa was created, the government banned the reception of migrants who intended to engage in low-skilled work—at least officially. These policies have resulted in a relatively small migrant population in the country. Indeed, the foreign population in Japan has grown almost threefold in the last three decades, reaching 2.9 million persons in 2019^4^; yet, migrants’ proportion of the population still remains a little over 2%. In this restrictive context, the Technical Intern Training Program (TITP) has served as Japan’s de facto guest worker program since its establishment in 1993. It has enabled government-designated industries and occupations to bring in and employ young adult migrants (called foreign technical intern trainees (TI trainees)) for a maximum of three (and five since 2017) years. These industries include agriculture, construction, food processing, and apparel and textile, where labor shortages are severe. With the aging and decline of the native-born population, TI trainees have increased in their number: 412,593 in 2019, a 170% growth from that in 2012.^5^ They mostly come from other Asian countries, with the Vietnamese accounting for over half of the total (53%) followed by the Chinese (20).^6^ Additionally, reflecting the nature of the program that TI trainees are called upon employers’ demand, they are now found in various parts of Japan; they were the most numerous among all foreign residents in 20 Japanese prefectures (of a total of 47) in 2019 (Oda & Fujisaki, 2020).

Besides low wages, studies suggest that an advantage of hiring TI trainees for businesses is to retain a “stable workforce” whose employment is secure and predictable (Kamibayashi, 2015). The TITP has the regulation that TI trainees cannot change worksites, which makes them such workforce, although the same rule often become a hotbed for exploitation. In fact, labor problems still seem prevalent among companies that use the program; a governmental inspection in 2019 revealed that 72% of TI-trainee-receiving workplaces violated some sort of labor-related laws, including one regarding work hours (22%), safety standard (21), and payment of extra wages (16) (though this figure include violations against Japanese employees too).^7^

Second, industrial characteristics of the U.S. and Japanese food processing sectors also differ. In the American industry, several giant meatpacking companies have developed and dominated the market, a tendency that continues to this day. According to the Government Accountability Office (2009), the four largest beef-processing, pork-processing, and poultry-processing firms have 79, 63, and 57% shares of total sales in the country, respectively. Some of the current major corporate actors are Tyson Foods, JBS USA, and Smithfield Foods (Telford & Kindly, 2020). Furthermore, the industry has grown amid greater employment of migrants; between 1990 and 2019, the total employment size increased from about 427,200 to 536,100 persons, a 25% growth.^8^ Contrastingly, the Japanese seafood processing industry primarily comprises small- and medium-sized companies; the aggregated annual sales of large seafood processing plants (those with over 300 employees) was less than 7% of the total sales in the industry in 2018.^9^ Moreover, this industry has been on a steady decline; between 1991 and 2016, the total employment decreased by 31%.^10^

Finally, an additional benefit of making the U.S.–Japan comparison concerns data availability. There has been an accumulation of research on the U.S. meatpacking industry and Latinx migration. I draw mainly on these studies to trace trajectories in which the industry has come to employ migrants. While research on the Japanese case is much more limited in the English-language literature, studies are available in the Japanese language, which I have familiarity with. I use them to specify the processes in the Japanese case. The analysis below begins with the U.S. meatpacking industry. It then moves on to examine the Japanese case, followed by the discussion comparing the findings.

## The U.S. meatpacking industry and labor migration

### Push factors from migrants’ traditional gateway states

Migration is a complicated and multifaceted process, which no single account can sufficiently explain (Massey et al., 1998). Latinx migration to the U.S. South and Midwest is no exception. In an early phase of this migration in particular, there were push factors at work that prompted migrants to leave from their traditional gateway states, which assisted their relocation elsewhere. One such factor is the implementation of an amnesty program to unauthorized migrants, legislated in the 1986 Immigration Act. Newly legalized, mostly Mexican migrants obtained freedom to move from old gateway states without fear of apprehension, which made it possible for them to pursue their economic fortunes elsewhere (Hernández-León & Zúñiga, 2000). The other policy factor involves the enforcement of the Mexico–U.S. border in the early 1990s. This caused geographic shifts in the crossing spots of unauthorized migrants away from old gateway states, discouraging potential migrants from relocating there once they successfully entered the region (Massey & Capoferro, 2008). A demographic factor was also at play, that is, the saturation of the migrant population in old gateways. This saturation caused the lowering of wages and the rise of rents for migrants living there, which facilitated their leaving (Light & von Scheven, 2008).

While these accounts tell us why Latinx migrants want to leave from traditional gateways, they fail to inform us about where they go instead. An account concerning the restructuring of the U.S. meatpacking industry fills this gap, suggesting how labor demand for migrants had been created in the rural Midwest and South by the 1990s.

### The restructuring of the meatpacking industry

As research on the restructuring of the meatpacking industry is relatively abundant (e.g., Broadway, 1995; Champlin & Hake, 2006; Huffman & Miranowski, 1996; Kandel & Parrado, 2005; Stanley, 1994; Schwartzman, 2013), here I highlight key points only. I also separately refer to the beef and pork sector and the poultry sector of the industry. Although both sectors have now become dependent on migrants, the trajectory of industrial development is somewhat different. An analysis starts with the beef and pork sector.

This sector underwent large-scale restructuring in the postwar period. Three processes of the restructuring are particularly relevant for the hiring of migrants in rural areas, including plant relocation, development of mass production, and deterioration of work conditions. Prior to restructuring, the beef and pork sector of the industry was among the earliest achievements of industrialization in the American industry. The labor historian Brody (1964) suggests that it “had been at the forefront of the mass-production revolution of the late nineteenth century” (p. 241). With the development of disassembly lines and minute division of labor, a handful of giant companies, the so-called Big Four, captured a large share of the market before the Second World War. These companies based their production facilities in Midwestern cities, notably, Chicago.

Principally initiated by companies newly established after the war, notably IBP (established in 1960 and acquired by Tyson in 2001), the industry underwent fundamental transformations. The first change is the plant relocation to rural areas in the Midwest (e.g., Iowa, Kansas, and Nebraska). Partly assisted by the establishment of interstate highways, this strategy brought an advantage of reducing transportation costs by building production facilities near to where livestock is raised (Broadway, 1995). By 2000, 60% of meatpacking jobs were located in rural areas (Kandel & Parrado, 2005).

The second change relates to the development of mass production. As noted above, the industry had already established this system in the pre-war period. Yet, the system saw further changes that produced an additional demand for low-wage workers. On one hand, there was increased automation. Owing to the nature of raw materials, which vary in shape and size, complete automation is difficult (Kandel & Parrado, 2005). Automation in this industry thus led to deskilling. In a cattle slaughter operation, for instance, power saws eliminated the demand for experienced cattle splitters (Horowitz, 1997). Moreover, with automation, plant size has expanded. From 1974 to 1997, the number of meatpacking plants employing more than 1000 workers doubled from 24 to 48 (Broadway, 2007). Also, in 1992, the share ratio of shipments by large plants (those with more than 400 employees) in the cattle- and hog-slaughtering industry, respectively, accounted for 72 and 86% of the total shipments (MacDonald et al., 2000). Large plants often have an enormous impact on the local labor market. For example, in the early 1980s, when IBP opened its plant near Garden City, Kansas, which was supposed to employ over 2000 employees, unemployment in the surrounding Finney county amounted to only 400 persons (Broadway, 2007).

The last structural change regards the deterioration of work conditions. The meatpacking job previously offered a decent wage, which was largely due to the establishment of an industrial union (the United Packing Workers of America) in the 1940s and its successful negotiation with the giant companies to gain a master contract. In 1969, for instance, the average wage of the meatpacking industry was 15% higher than the overall manufacturing wage average (Stanley, 1994). Significant wage decline ensued, however, with decreasing union influence. Following IBP’s strategy, especially since the 1980s, a number of companies have refused to conclude the master contract, with strategies including forcing the union to accept concessions, closing unionized plants, and newly opening non-unionized plants (Horowitz, 1997). The decline in union representation led to wage reductions, and by 2002, meatpacking wages were 25% below the manufacturing average (Broadway, 2007). In addition, the decline of unionism accompanied an increase in the speed of the disassembly line, and the incidence of occupational illnesses grew by 264% between 1980 and 1988 (Horowitz, 1997). In 1992, the injury rate of the industry was 30%, and although it declined to 15% in 2000, the figure is still among the highest in the American industry (Government Accountability Office, 2005). The worsening of work conditions has, in turn, resulted in worker turnover. Estimates vary between studies, but they commonly point to high turnover rates, including 6–8% per month (Broadway, 2007) and 60–140% or possibly higher annually (Kandel & Parrado, 2005).

In the poultry sector, the path of industrial development is somewhat different. One difference is that industrialization occurred late; for most part of the pre-war period, the processing of chicken was a byproduct of egg production practiced by small farmers (Stull & Broadway, 2004). Only in the 1950s did the poultry production begin its large-scale industrialization, establishing a high degree of vertical integration that involved hatching, growing poultry, feed supply, and processing (Boyd & Watts, 1997). Another difference is that there has been no significant plant relocation. Since the inception of industrialization, rural areas of the Southern states have been principal production sites. In 2002, the top five states in terms of the number of employees in the poultry sector were Arkansas, Georgia, North Carolina, Alabama, and Mississippi, counting 52% of all employees in the country (Champlin & Hake, 2006). Boyd and Watts (1997) provide three reasons as to why the sector initially concentrated in this region: the presence of small marginal farmers to be contracted as poultry growers, of merchants and feed dealers who extended credit to these farmers, and of surplus rural labor available to the processing facilities. The last difference is that the sector has not experienced unionization and wage improvements comparable to those that occurred in the beef and pork sector. Located in the rural South, this sector has been a low-wage business since its establishment as an industry. Before the massive employment of migrants, the sector had largely drawn on African Americans to comprise an inexpensive workforce (Boyd & Watts, 1997; Schwartzman, 2013).

Despite these differences, there is also similarity to the beef and pork sector. The first is an establishment of large-scale mass production. For example, the share ratio of shipments by large poultry plants rose from 29% of the total in 1967 to 88% in 1992 (MacDonald et al., 2000). Schwartzman (2013) suggests that today’s poultry processing facilities are “factories with mechanized high-speed line” where the “technology required constant attendance” (p. 61). In addition, with the development of mass production, the nature of work has become dangerous. Stuesse (2016) maintains that the “production [has been] sped up through remarkable technological advances, and workers now repeat the same monotonous—and often hazardous—movement throughout their entire shift” (p. 6). In 2000, the injury statistics from the Occupational Safety and Health Administration showed that one out of every seven poultry workers was injured on the job (Stein, 2002).

To summarize, although the development of the beef and pork and the poultry sectors was not identical, these two sectors similarly went through the development of mass production and the worsening of work conditions, which resulted in producing large labor demand for low-paid and hazardous work in rural areas by the 1990s. This transformation led to the employment of migrants. Although the work in the industry may now be unattractive to the local native-born, it is appealing to migrants, especially those with low educational credential and/or limited English proficiency. Through interviews with Latinx migrants in three meatpacking communities in Nebraska, Dalla et al. (2005) suggest that they were primarily attracted to the meatpacking work as it offers a stable, year-round employment with a relatively good pay, at least in comparison with field labor. An attraction for a higher wage rate than the one in migrants’ traditional gateways is also echoed by Huffman and Miranowski (1996). The interviews also indicate that, for those with a family, “peaceful” rural life and a greater chance for purchasing home, as compared with the life in an inner city, were also attractive. Indeed, despite the high turnover in the industry, some migrants seem to have settled in rural meatpacking communities (Griffith, 2019).

### Corporate recruitment and migrant social networks

Labor demand for migrants does not automatically bring them to rural meatpacking towns. There have been two social processes on the ground. One is the corporate recruitment of migrants, conducted in border towns, migrants’ concentrated areas, or their home country (Broadway, 2007; Champlin & Hake, 2006; Stuesse, 2016). Companies used this method especially in an initial phase of migration, but, once migration is initiated, the other process is usually set in motion. It is migrants’ mobilization of their social networks, the importance of which a bulk of the immigration literature has so far stressed (Massey et al., 1998). In the case of the meatpacking industry, Grey and Woodrick (2002) find that, in Marshalltown, Iowa, more than half of the Mexican employees in a local meatpacking plant came from the same Mexican community. In his study on poultry plants in Georgia, Griffith (1995) also reveals that about 86% of the new workers had been recruited through friendship or kinship ties. To meet labor demand for jobs with high turnover, this recruitment is often encouraged by employers, who pay bonuses to their workers who bring new ones to the plant (Broadway, 2007; Griffith, 1995; Stanley, 1994).

Two more considerations should be made before concluding this section. The first is that migrants have not necessarily increased in this industry in such a straightforward manner as the social network explanation often implies. These migrants are often unauthorized, and there have occasionally been high-profile immigration enforcements, such as the raids in six Swift & Co. plants in 2006, which recorded 1300 arrests to be the largest single worksite raid in U.S. history. When these crackdowns occurred, the reduction in the share of the unauthorized in the industry followed. Yet, the effect seems to last temporarily (Groves & Tareen, 2020), with the still strong labor demand for migrants, including the unauthorized. This was partially revealed in 2019 when large-scale immigration raids were implemented against seven poultry plants in Mississippi; they recorded nearly 700 arrests to be the largest statewide immigration crackdown in the history (Fausset, 2019).

Second, while the hard and dangerous meatpacking work initially created the demand for migrants, it is plausible that, as migrants’ social networks ensure the continued availability of migrants, this availability has in turn contributed to preserve such work. Although proving this relationship with data is not easy, what happened to Crider Inc., a poultry processor in Georgia, may offer a hint. The company lost three-quarters of its workforce when the immigration agents visited its plant in 2006. While the company responded by raising wages and recruiting local African American workers afterward, disputes and high turnover followed because of what these workers saw as low pay and poor work conditions. This prompted the president to say that “he preferred Hispanics because ‘We want people who want to work and are willing to work every day’” (Rural Migration News, 2011).

## Migration in the Japanese seafood processing industry

### The impact of the TITP

The Japanese seafood processing industry has grown in geographic proximity to its fisheries, whose base is scattered along coastal areas of the country. Major fishing ports are often located outside large cities. For instance, compare the geographic location of Japan’s 13 “specified type III fishing ports” (those that are designated by law as being of particular importance for the promotion of the fishing industry) with that of 20 “ordinance-designated cities” (those with a population of at least 500,000 people and designated by the central government to have greater political autonomy; a proxy of major large cities): There is only one case (Fukuoka City) in which these two designations overlap.

**Fig. 1:**
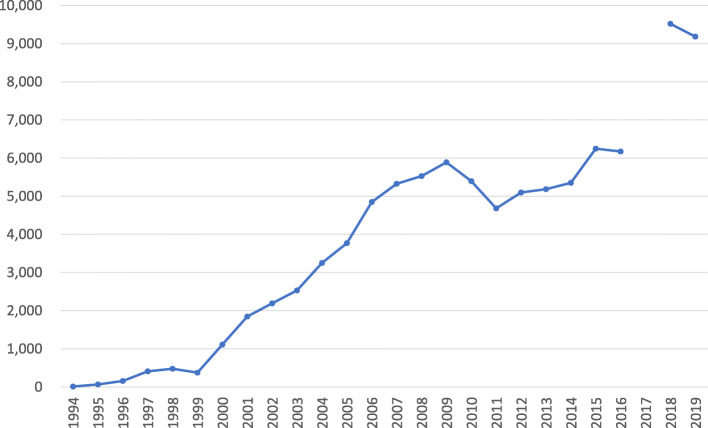
The Number of First-Year Technical Intern Trainees Employed by the Japanese Seafood Processing Industry (1994–2019). Notes: Numbers from 1994 to 2016 are from the Japan International Training Cooperation Organization (2000-2018), and those from 2018 to 2019 are from the Organization for Technical Intern Training (2019-2020). Owing to different counting methods, data from these two sources are not directly comparable. A number for 2017 is not shown because complete data are not available. Source: Created by the author based on the Japan International Training Cooperation Organization (2000-2018) and the Organization for Technical Intern Training (2019-2020) data

Although geographic locations have changed little, the workforce composition has been transformed with the increase of migrants. One key factor is the TITP. In this industry, the employment of TI trainees has been accelerated since 2000, when two major occupations—the production of heated and unheated seafood products—were added for the use of this program by the government. The number of TI trainees that the industry annually receives shows a steady increase since that year, except for a brief hump after the 2008 recession (Fig. 1). In 2019, in accordance with the general trend, 55% of TI trainees in the seafood processing industry were Vietnamese, followed by Chinese (27) (Organization for Technical Intern Training, 2019-2020).^11^

To be sure, that the seafood processing industry wants migrants is not overtly surprising, given that it is susceptible to shortages of native-born workers. The work in this industry is often unpleasant dealing with raw fish. The necessity of cutting tasks also makes the work no less safe than one in other industries.^12^ Moreover, this industry provides low wages, which have hitherto been at or near the hourly minimum. Perhaps as both cause and result of low wages, the industry has traditionally relied upon a specific segment of the native-born, that is, local women (particularly stay-at-home mothers), whom it hires with the precarious status of part-time employees (Miki, 2005). Yet, recruiting these women has proven difficult more recently, because of the aging and decline of the population, which is Japan’s recent demographic feature but is particularly severe outside large cities, as well as an increase in alternative job opportunities for them (e.g., cashier work at supermarkets) (Miki, 2005; Sasaki, 2014).

While undoubtedly important, it is important to note that this line of reasoning does not fully explain the process through which the industry has depended on migrants. This is the case considering that not all seafood processors apparently want them, which becomes clear as we see geographic variations in the degree to which the local seafood processing industry employs migrants. Using the 2013 Census of Fisheries, Mazumi (2019) found that there were no migrant workers in the seafood processing industry in 254 of the 536 municipalities (cities, towns, or their equivalents) where this industry was present in Japan; meanwhile, they made up for over 15% of the local seafood processing workforce in 48 municipalities, with a national average of 7%. This implies that factors other than the TITP are also important.

### Establishing mass production in the seafood production

One such crucial factor is the establishment of mass production. As aforementioned, Japanese seafood processors are largely small or medium in size. Nevertheless, some of them have developed mass production—albeit limited in scale—in the postwar years, as the nature of business shifts from seasonal to year-round operation owing to the development of refrigerating technology and fishery techniques, the use of imported materials, and so on. These processors are more likely to employ TI trainees because, as the Japanese migration literature suggests (Kamibayashi, 2015), they represent the stable workforce, whose employment security and predictability are necessary for the working of mass production.

While Japanese seafood products are diverse, the production of dried bonitos (*katsuobushi*) offers a symbolic illustration in this regard. Dried bonitos are a popular food in Japan, and though they are traditionally used to make broth, they are also sold as thin flakes to flavor other foods. In 2000, the production volume of dried bonitos accounted for over 40,000 tons, a large increase from the 14,360 tons recorded in 1969 (Shiragai, 2004); this number becomes even more significant considering that most seafood products have reduced their production in the country. A direct cause for this growth can be traced back to 1969; in this specific year, Ninben, an old-established food processor located in Tokyo, invented what is now called a “bonito pack” (*katsuo pakku*), which comprises thin flakes of dried bonitos in small packages. The product became a big hit in the market (Miyauchi & Fujibayashi, 2013; Shiragai, 2004), and other food processors followed suit to capture a share of this expanding market; meanwhile, they specialized in slicing and packaging, leaving the dried bonito production to smaller-sized seafood processors.

As de facto or de jure subcontractors, dried bonito processors came to develop a mass-oriented system. Two transformations are noteworthy. The first relates to changes in the type of finished product. Processing companies traditionally spent weeks fermenting their raw materials to achieve the final product (*shiagebushi*); however, they abandoned this process to shorten their lead time for slicing, instead focusing on dried bonitos that do not undergo fermenting (*arabushi*) (Miyauchi & Fujibayashi, 2013). The second involves automation. Since the nature of raw materials hinders the level of standardization that can be achieved, the production process requires repetitive manual work such as cutting, deboning, and stacking bonitos in the smoking room (Sasaki, 2014). Still, there are some limited instances of automation and deskilling, which include the invention of head-cutting machines, the automation of the boiling process, the installation of conveyers in the deboning process, and the introduction of smoking machines with fanning functions (Kataoka & Mantjoro, 2008; Ounabara, 2006; Shiragai, 2004).

This development necessitates the year-round availability of full-time workers. Nonetheless, it has increasingly become difficult to secure such labor locally, with one primary reason being population aging and decline. For instance, in Makurazaki City (Kagoshima), one of the major sites of dried bonito production, the local population decreased to 23,638 in 2010 from about 30,000 in the 1980s, with a local elderly rate of 32% (Sasaki, 2014).

The need for a workforce that supports mass production led to the hiring of TI trainees (Kataoka & Mantjoro, 2008; Sasaki, 2014). Although it is difficult to accurately indicate the degree of dependence on migrants of dried bonito processors, this can be inferred by considering three major sites for dried bonito production: Yaizu City (Shizuoka), Makurazaki City (Kagoshima), and Ibusuki City (Kagoshima) (Miyauchi & Fujibayashi, 2013). In 2018, the share of migrants in the seafood processing industry of these municipalities was about 14% (out of 3625 persons), 23% (out of 1144), and 18% (out of 639), respectively, which are figures above the national average (10%).^13^

The significant association between mass production and demand for TI trainees can also be illustrated from a statistical angle. Mazumi (2019), using the 2013 Census of Fisheries, examine local variations for migrant employment ratios in the seafood processing industry. The study reveals that, controlling for other factors (including the relative employment size of the industry in the local workforce), higher productivity in the local seafood processing industry was associated with a higher migrant employee ratio.

## Discussion

Japan and the United States are two developed countries where, during the last few decades, the hiring of migrants in the food processing industry has contributed to migration flow outside large cities. Against this backdrop, this study delved into the commonality governing how the two countries’ industries have developed a dependence on migrants. Table 1 summarizes the processes present in the United States in comparison with the Japanese ones. As shown in the table, overall, the processes are significantly different on the two sides of the Pacific. In the U.S. case, the post-war transformation of the meatpacking played a central role. Although it was somewhat different depending on the sectors, the industry witnessed the development of large-scale mass production and, concomitantly, the work conditions in rural areas deteriorated. While these changes made the work of this industry unappealing to the native-born, they attracted migrants with low education and/or English proficiency for what they perceived to be better conditions than agricultural work and, for some, living environments in rural towns. Migrants were initially recruited by companies, but, once job opportunities were found, their social networks were strengthened to bring in more migrants, which replenished workers for enabling large-scale, continuous production despite hazardous work.

**Table. 1:** Processes that Led to the Hiring of Migrants in the Food Processing Industry in the United States and Japan

	Push factors		Processes on industrial restructuring			Corporate recruitment and migrant social networks	Outcome
	Policy that discouraged migrants to stay in major gateways	Migrant saturation in major gateways	Plant relocation	Development of mass production	Deterioration of work conditions		
The U.S. beef and pork sector	Yes	Yes	Yes (mainly from Midwestern cities to Midwestern rural areas)	Yes (large-scale mass production further enhanced)	Yes (regarding both wage level and worksite injuries)	Yes	The hiring of migrants
The U.S. poultry sector	Yes	Yes	No (industry developed in the Southern rural areas)	Yes (large-scale mass production established)	Yes (regrading worksite injuries; wage level remains low)	Yes	The hiring of migrants
Japanese seafood sector	No (rather, there is the TITP as an important policy)	No	No (industry developed in coastal cities and towns)	Yes (mass production established, albeit limited in scale)	No (rather, external changes involving local demography and labor market matter)	No (migrant hiring is regulated by the TITP framework)	The hiring of migrants

*U. S.* United States, *TITP* Technical Intern Training Program

Japan’s case is apparently very different. One significant difference is that, in the context of the restrictive legal context of migration, the creation of the TITP provided a direct impetus for seafood processors to employ migrants. Additionally, these seafood processors are much more limited in size and resources as compared with their American meatpacking counterparts; thus, the former has not experienced industrial transformations in a degree that is comparable to the latter. However, despite these differences, there is also commonality, which is the development of mass production. Though the scale of production differs, some Japanese seafood processors have established mass production in the second half of the last century, and they have become more likely to hire TI trainees. For the functioning of mass production in the Japanese seafood processing industry, as well as its U.S. meatpacking counterpart, an undisrupted availability of full-time as well as low-wage workforce is necessary. In the context of the declining availability of the native-born as such workers, seafood processors turned to TI trainees as an alternative workforce.

A caution is needed as to the way that migrants are mobilized for ensuring such availability. This mobilization can be different between the two countries due to the difference in legal and institutional contexts of migration. On one hand, American meatpackers may be relying on what can be called the *group stability* of migrants, namely, the continuous hiring of many individuals. On the other hand, Japan’s seafood processors draw on the migrant *individual stability*, which refers to individuals’ continued employment, made possible by the TITP’s regulation that prohibits TI trainees from changing worksites.

Regardless, the fact remains that mass production of the food processing industry has been enabled by the hiring of migrants on both sides of the Pacific. Thus, this study concludes that the development of mass production led to the employment of migrants in the food processing industry, which explains a partial but important part of labor migration outside large cities both in Japan and the United States.

### Addressing robustness

How common is an association between mass production and labor migration beyond the United States and Japan? If this association has robust cross-national generalizability, it should be observed beyond these two cases as well. Although relevant research is limited in other national settings, the available evidence still indicates that this association can be generalized beyond the United States, Japan, North America, and East Asia.

An example is the British meatpacking industry. Since the EU expansion in 2004, its dependence on migrants (especially from Eastern Europe) has increased. According to the Migration Advisory Committee (2018), 69% of the workforce in the meat processing sector is comprised by EU27 migrants. Importantly, the role that migrants assume in Britain is not entirely identical to that in the United States or Japan. Existing research tends to note the flexible role of migrants in Britain, many of whom are referred to as agency workers, who are hired and dispatched by temporary work agencies (James & Lloyd, 2008; Lever & Milbourne, 2017; Tannock, 2015). In Britain, a handful of giant supermarkets exert considerable market power, so the meatpacking industry is subject to sudden changes in their order volume. In this context, migrant workers function as a workforce that can be on the employers’ immediate disposal.

Despite this specificity, mass production seems to be linked to the hiring of migrants in Britain. First, mass production characterizes the basic system of this industry in the nation. According to James and Lloyd (2008), “[a] utomated, continuous production processes … are prevalent within much of the [the British food processing] industry, and a high proportion of companies produce relatively simple, standardized products” (p. 211). The St. Merryn Meat factory, in the small city of Merthyr Tydfil, Wales, which is “regularly touted as being one of the largest and most high-tech meat-processing facilities in Britain” (Tannock, 2015, p. 6), may serve as an example. By the 2000s, over 61% of its 1016 workers were migrants (Tannock, 2015). Second, the significant presence of migrants also appears among non-agency workers. Equality and Human Rights Commission (2010), a British non-governmental public body, reports that over one-third of the permanent workforce in the industry are migrants.^14^

The Norwegian seafood processing industry offers another illustration. Located along rural coastal areas, its reliance on migrant workers from the EU has grown since the EU expansion in 2004, like the case of Britain.^15^ By 2013, 48% of the seafood processing workforce was comprised by migrants nationally (Friberg & Midtbøen, 2019). This recent demand for migrants is partly connected to the development of fish farming, which has made a stable supply of raw materials possible, creating a year-round demand for manual labor as well as facilitating mechanization in processing operations.

The experiences of the small neighboring municipalities of Hitra and Frøya provide a symbolic example. Since 2005, fish farming has undergone an expansion in the region, which now hosts the production facilities of three national leading companies. According to Rye (2018), notable among them is SalMar’s “new state-of-art high-tech fish processing facility at Frøya” (p. 194). By 2018, about 1730 migrants resided in the region, making up for 19% of the total population, a large increase from the 2005 population (281 migrants) (Rye, 2018).

In summary, the above European cases, together with the U.S. and Japanese cases, confirm that the development of mass production is a defining characteristic of transformation of the food processing industry that is associated with the recent growth of the migrant population outside large cities. This insight is important. In explaining labor migration in light of structural transformations of advanced economies, the urban-centered migration research often emphasizes the rise of low-wage services or “downgraded,” small-batch manufacturing as an economic driving force of migration. However, this study demonstrated, with cross-national generalizability, that a different process of industrial transformations exists to create the recent migration flow outside large cities.

## Conclusion

This study has two contributions. First, while previous research on the food processing industry and labor migration is largely oriented to a single-national case, this study engaged in an international comparative study. By so doing, it found that the development of mass production was the common process that resulted in labor migration outside large cities. Second, by identifying the significance of mass production, the study also suggested the difference in the pattern of industrial transformations that is associated with labor migration between in and outside large cities.

One important limitation of this study is that, though significant, the food processing industry is not the sole business that relies on migrants in non-large cities. This poses a question regarding the generalizability of mass production beyond the case at hand. Space limitations preclude sufficiently addressing this issue here. Yet a brief look at other notable cases indicate that mass production may equally matter. One instance is agriculture, which has recently resulted in large migration flow especially in rural Europe (Rye & Scott, 2018). McAreavey and Argent (2018) suggests that the restructuring of agriculture for greater economies of scale, which requires “a flexible and steady supply of low wage labour” (p. 149) to fill both seasonal and full-time positions, is relevant for this trend. Rye and Scott (2018) also relate this migration with “a shift away from family towards industrialised farming” (p. 930). As for manufacturing, the case of the carpet industry in the American rural South, especially the state of Georgia, which accounts for the majority of products made in the country, may serve as an example. With industrialization occurred in the last century, the industry has developed to employ Mexican migrants. Hernández-León and Zúñiga (2000) suggest that these migrants “are overwhelmingly employed in labor intensive stages” (p. 58) of the production process, engaging in low-skilled work. These examples provide an additional credence on the central role of mass production in creating labor migration outside large cities. In essence, however, further research is warranted to highlight this issue.

## Data Availability

All data are available from the sources quoted in this article.

## Footnotes

1 Data from the Ministry of Health, Labour and Welfare, available at: https://www.mhlw.go.jp/stf/newpage_16279.html.

2 Data calculated by the author, using the 2018 Census of Fisheries, which is available from e-Stat at: https://www.e-stat.go.jp/stat-search/files?page=1&toukei=00500210&tstat=000001033844&cycle=0&year=20180&month=0&tclass1=000001132724&tclass2=000001136323.

3 Note that these Latinxs include the native-born as well. Hereafter, a note will be made when, due to the nature of data that I refer to, it is unable to distinguish the foreign-born from the native-born.

4 Unless otherwise noted, statistical data in this paragraph are from the Ministry of Justice, available at: https://www.e-stat.go.jp/stat-search/files?page=1&layout=datalist&toukei=00250012&tstat=000001018034&cycle=1&year=20190&month=24101212&tclass1=000001060399.

5 This tendency stands in sharp contrast to *Nikkeijin* (Japanese-origin migrants), another source of de facto migrant workers, whose population has declined or stagnated since the 2008 recession.

6 The Vietnamese surpassed the Chinese in the share in 2016. The absolute number of Chinese TI trainees has also recently been declining, perhaps because of growing economic opportunities at home.

7 Data from the Ministry of Health, Labour and Welfare, available at: https://www.mhlw.go.jp/stf/newpage_13980.html.

8 Data from the Bureau of Labor Statistics (animal slaughtering processing), available at: https://www.bls.gov/data/.

9 Data calculated by the author, using the 2018 Census of Fisheries. See footnote 2 for source.

10 Data from the 2016 Economic Census (seafood products), available at: https://www.e-stat.go.jp/stat-search/database?page=1&toukei=00200553&tstat=000001095895, and the 1991 Establishment Census of Japan, available at: https://www.e-stat.go.jp/stat-search/database?page=1&toukei=00200551&tstat=000000000095.

11 Statistics on the gender make-up in the industry is not available, but existing case studies indicate that the majority of TI trainees in the industry were women (Iwasa, 2008), which, as mentioned immediately, perhaps reflects the traditional pattern of employment in the industry.

12 This can be partially glimpsed from a result of the 2019 governmental inspection targeted at TI-trainee-receiving workplaces. Among all labor-related-law violations found for food processors, the most frequent (28%) was concerning safety standard. See footnote 7 for source.

13 Data calculated by the author, using the 2018 Census of Fisheries. See footnote 2 for source.

14 What will occur in the post-Brexit era, of course, is a different issue.

15 Norway is not a part of the EU, but a member of the European Economic Area (EEA).
